# Comparative analysis of adaptive and neutral markers of *Drosophila mediopunctata* populations dispersed among forest fragments

**DOI:** 10.1002/ece3.4696

**Published:** 2018-11-22

**Authors:** Marcos R. D. Batista, Rafael E. S. Penha, Silvia H. Sofia, Louis B. Klaczko

**Affiliations:** ^1^ Departamento de Genética, Evolução, Microbiologia e Imunologia Instituto de Biologia, Universidade Estadual de Campinas – Unicamp Campinas SP Brasil; ^2^ Departamento de Biologia Geral, Centro de Ciências Biológicas Universidade Estadual de Londrina Londrina PR Brasil

**Keywords:** cline, gene arrangements, genetic diversity, microsatellites, tripunctata

## Abstract

Comparison of adaptive and neutral genetic markers is a valuable approach to characterize the evolutionary consequences of populations living in environments threatened by anthropogenic disturbances, such as forest fragmentation. Shifts in allele frequencies, low genetic variability, and a small effective population size can be considered clear signs of forest fragmentation effects (due to genetic drift) over natural populations, while adaptive responses correlate with environmental variables. Brazilian Atlantic Forest had its landscape drastically reduced and fragmented. Now, several forest remnants are isolated from each other by urban and crop areas. We sampled *Drosophila mediopunctata* populations from eight forest remnants dispersed on two adjacent geomorphological regions, which are physiognomic and climatically quite distinct. Microsatellite data of inversion‐free chromosomes (neutral genetic marker) indicate low structuration among populations suggesting that they were panmictic and greatly influenced by gene flow. Moreover, significant differences in chromosomal inversion frequencies (adaptive genetic marker) among populations and their correlations with climatic and geographical variables indicate that genetic divergence among populations could be an adaptive response to their environment. Nonetheless, we observed a significant difference in inversion frequencies of a population in two consecutive years that may be associated with edge and demographic effects. Also, it may be reflecting seasonal changes of inversion frequencies influenced by great temperature variation due to edge effects. Moreover, the forest fragment size does not affect genetic variation of neutral markers. Our data indicate that despite oscillations in chromosomal inversion frequencies, *D*. *mediopunctata* populations from Brazilian Atlantic Forest and their divergence may be driven by adaptive factors to local differences, perhaps because it is a small flying insect easily carried by the wind increasing its migration rates.

## 1 INTRODUCTION

Changes of natural landscape in different ecosystems around the world caused by forest fragmentation processes may be very harmful to biodiversity (Millette & Keyghobadi, 2015; Newmark & McNeally, 2018) and may affect the genetic structure of populations (Radespiel & Bruford, 2014; Rhoads, Williams, & Krane, 2017; Rosche et al., 2018). An appropriate approach to infer the nature of genetic responses in stressful environments is to compare population parameters such as number of migrants per generation, fixation index, and allele frequencies shifts using adaptive and non‐adaptive genetic markers (Hoffmann & Willi, 2008; Merilä & Hendry, 2014; Stojanova et al., 2018).

Chromosomal inversion polymorphisms have been studied for a long time and are usually under some form of balanced selection (for detailed revisions see: Garcia & Valente, 2018; Hoffmann & Rieseberg, 2008; Powell, 1997). They allow insights on the action of natural selection in both natural and laboratory populations by monitoring inversion frequency shifts (Dobzhansky, 1947; Dobzhansky & Levene, 1948). These shifts in natural populations are often associated with seasonal (Wellenreuther, Rosenquist, Jaksons, & Larson, 2017) and long‐term variation (Batista, Ananina, & Klaczko, 2012; Etges, Arbckle, & Levitan, 2006; Orengo, Puerma, & Aguadé, 2016). Similarly, assessing geographical variation one can unveil patterns, which might be interpreted as prima facie evidence of natural selection (Ayala et al., 2017; Simões, Calabria, Picão‐Osório, Balanyà, & Pascual, 2012).

Microsatellite loci are codominant multi‐allelic genetic markers, which allow assessing both temporal and spatial genetic structure of natural populations (Gredler, Hish, & Noor, 2015; Hartvig et al., 2018; Silva, Machado, & Mateus, 2015). They are also a marker commonly used in conservation genetics to estimate the loss of genetic variability and to infer the demographic history of populations, assuming they are neutral or nearly neutral even if located in a coding region (Ellegren, 2004; Lombaert et al., 2018; Stamenković‐Radak et al., 2012; Takezaki, 2017).

Forest fragmentation and deforestation are believed to make environmental conditions more heterogeneous, with pronounced changes in biotic and abiotic conditions (Keyghobadi, 2007). Stochastic shifts in frequencies of genetic markers and high divergence among populations are expected to be the major responses of populations from a fragmented landscape (Milligan et al., 2018; Schippers et al., 2015).

Currently, the Atlantic Rainforest biome in Brazil is extremely fragmented (Joly, Metzger, & Tabarelli, 2014). About 80% of its forest remnants encompass areas smaller than 50 ha (Ribeiro, Metzger, Martensen, Ponzoni, & Hirota, 2009). Many of its forests remnants are scattered amid pastures, agricultural fields and growing urban landscape, especially in the states of São Paulo, Rio de Janeiro and Minas Gerais (Joly et al., 2014). There are more than a hundred forest fragment remnants in the city of Campinas, which is 61 km north of the Tropic of Capricorn (Cielo‐Filho & Martins, 2016; Cielo‐Filho, Gneri, & Martins, 2007). Although all forest remnants from this region can be classified as Seasonal Semi‐deciduous Forests, various studies have shown that they are heterogeneous (Cielo‐Filho & Martins, 2016; Cielo‐Filho et al., 2007; Salis, Shepherd, & Joly, 1995). Furthermore, they are located over an ecotone in the transition area between two geomorphological regions (GMRs): Peripheral Depression and Atlantic Plateau (Joly et al., 2014; Ross, 2013).

Marked differences are observed between these two GMRs. Considering climatic variables such as average monthly temperature and annual sum of monthly precipitation, they differ in approximately 1°C and more than 70 mm, respectively (Table 1; see also Alvares, Stape, Sentelhas, Moraes Gonçalves, & Sparovek, 2013). In relation to their geological properties, Peripheral Depression unit is characterized by a flat topography and lands of magmatic sedimentary rocks with crystalline rocks; and Atlantic Plateau is characterized by orogenic belts, a continuous range of mountains with deep valleys and channels, with several soil types including cambisols, lithic, podzolic and podzolic yellow‐red and red‐yellow oxisol, and rocky outcrops (Ross, 2013).

**Table 1:** Forest remnant geographical and climatic variables

GMR	Site	Latitude	Longitude	Altitude (m)	Area (ha)	Temperature (°C)	Precipitation (mm)
Peripheral depression	CV	23°03′S	47°28′W	530	15	22.0	1,144
	SG	22°49′S	47°07′W	605	250.4	22.3	1,411
	CS	22°52′S	47°04′W	600	14.3	22.3	1,413
Atlantic plateau	PE	22°55′S	47°01′W	675	3.5	21.6	1,488
	CA	22°50′S	46°56′W	650	244.9	21.0	1,487
	IT	22°27′S	44°37′W	950	30,000	19.7	1,830
	JF	21°45′S	43°19′W	860	277	20.6	1,402
	TE	22°27′S	43°00′W	1,140	24,024	19.9	1,316

Note

CV: Capivari; SG: Santa Genebra; CS: Costa e Silva; *GMR*:* geomorphologic* region; PE: Parque Ecológico; CA: Colinas do Atibaia; IT: Itatiaia; JF: Juiz de Fora; TE: Teresópolis.

**Figure 1:**
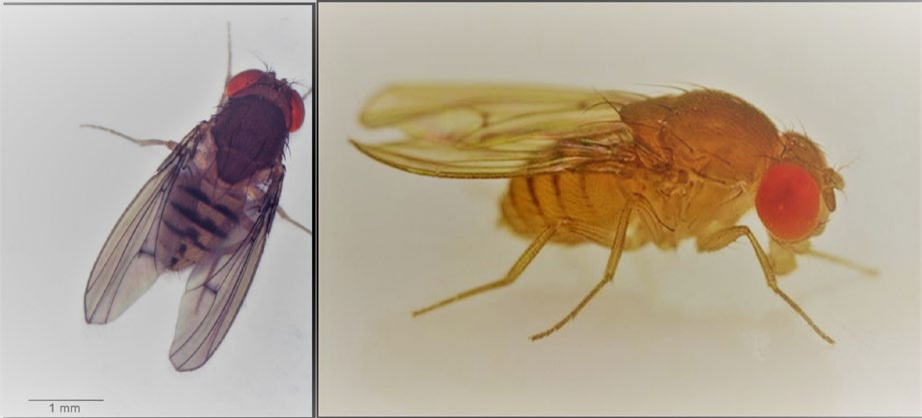
Photographs of *Drosophila mediopunctata*. Left photograph: Marcos R. D. Batista; right photograph: Gustavo M. Mori

*Drosophila mediopunctata* (Figure 1) is found in winter in good numbers in these two GMRs, as well as in many other places of the Atlantic Rainforest biome, especially in Southern Brazil and in high altitudes (Batista, Rocha, & Klaczko, 2018; Saavedra, Callegari‐Jacques, Napp, & Valente, 1995). This is an almost exclusively forest‐dwelling Neotropical species belonging to the *tripunctata* group, subgenus *Drosophila* (Bächli, 2018; Hatadani et al., 2009; Vilela, 1992; Yotoko, Medeiros, Solferini, & Klaczko, 2003).

This species has five pairs of acrocentric and one pair of dot chromosomes (2*n* = 12) (Ananina, Peixoto, Souza, & Klaczko, 2002; Kastritsis, 1966). It has good polytene chromosomes and is highly polymorphic for chromosome inversions (Klaczko, 2006). Chromosomes II, IV, and X are polymorphic for inversions (with 17, two and four gene arrangements, respectively) while chromosomes III and V are inversion‐free (Ananina et al., 2002; Brianti, Ananina, & Klaczko, 2013). Thus, to avoid biases on the genetic structure estimates by hitchhiking effect due to selection on chromosome inversions (Kennington & Hoffmann, 2013; Santos et al., 2016), one may use microsatellite loci located in the inversion‐free chromosomes (Cavasini, Batista, & Klaczko, 2015; Laborda, Gazaffi, Garcia, & Souza, 2012). On the other hand, adaptive responses can be inferred from chromosome II inversion polymorphism, which is associated with variation in other traits that affect fitness, such as size and shape of the wing and genitalia and polychromatism (Andrade, Vieira, Ananina, & Klaczko, 2009; Bitner‐Mathé, Peixoto, & Klaczko, 1995; Hatadani & Klaczko, 2008; Hatadani, Baptista, Souza, & Klaczko, 2004). Furthermore, chromosome II inversion polymorphism shows correlation with climatic variables (temperature and precipitation) congruent with an altitudinal cline and seasonal cycling variation described for the natural population from Parque Nacional do Itatiaia, Rio de Janeiro State (Ananina et al., 2004; Batista et al., 2012; Klaczko, 2006).

Ananina et al. (2004) studying various populations of *D. mediopunctata* in a geographic transect found a puzzling result. In spite of their geographic distance, two natural populations showed stark differences in chromosome II inversion frequencies. Santa Genebra population, located in Peripheral Depression, showed clear differences in chromosome II inversion frequencies when compared to Japi population. These two natural populations are distant about 50 km. On the other hand, Japi had frequencies similar to Itatiaia, which is about 250 km distant. Interestingly, both Japi and Itatiaia are located on the same GMR, Atlantic Plateau.

Two simple alternative scenarios (adaptive vs. neutral) can be advanced to explain this finding and other chromosomal inversion frequency geographical differences. In the adaptive case, we expect populations to be panmictic with low genetic structure (high gene flow) and geographical differentiation of chromosomal inversion frequencies correlated to each GMR. Under the neutral case, the observed difference is a casual oscillation caused by forest fragmentation (genetic drift), and we expect to observe populations highly isolated (great genetic structure and low gene flow), great level of linkage disequilibrium in neutral markers and stochastic shifts in chromosomal inversion frequencies (with no pattern).

**Figure 2:**
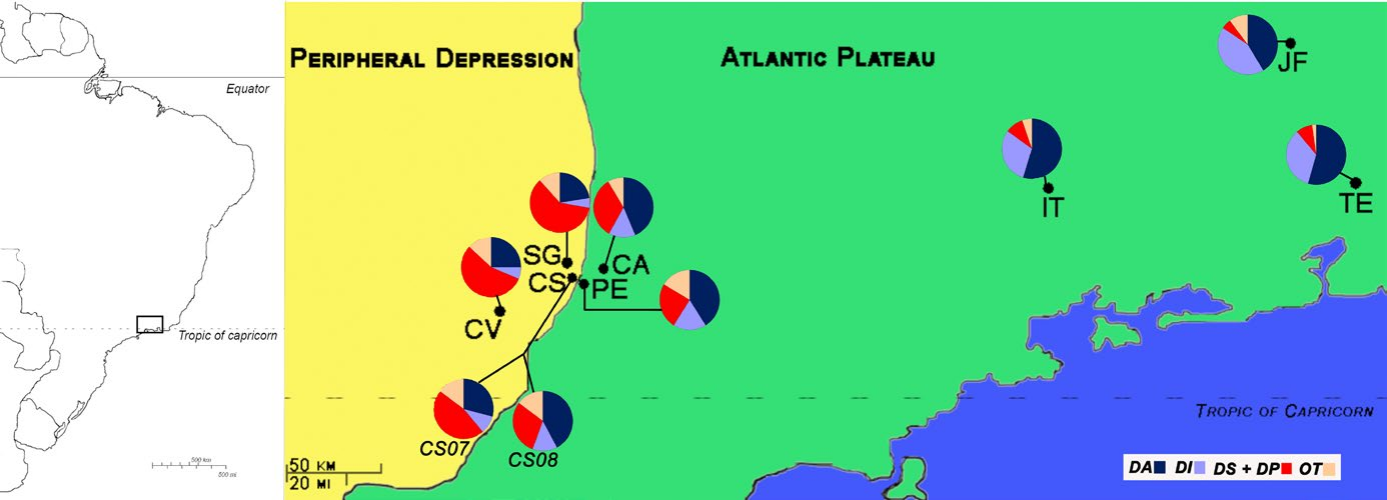
Map of the studied region and the distribution of inversion frequencies for each collected site: CV: Capivari (SP); SG: Mata Santa Genebra (Campinas—SP); CS: Costa e Silva (Campinas—SP); PE: Parque Ecológico (Campinas—SP); CA: Colinas do Atibaia (Campinas—SP); IT: Parque Nacional do Itatiaia (Itatiaia—RJ); JF: Juiz de Fora (Reserva Municipal do Poço D'Anta—MG); TE: Teresópolis (RJ—Parque Nacional das Serra dos Órgãos). Figure drawn based on the map published by Ross, 2013. Note: for Costa e Silva population (CS), there are two graphs corresponding to the two collections carried out (CS07 and CS08)

To test them, we sampled populations in the Campinas Area (Campinas city and Western nearby Capivari town) and in the Eastern Area (Itatiaia and Teresópolis in Rio de Janeiro State; and Juiz de Fora in Minas Gerais State; see Figure 2). Furthermore, we contrasted two kinds of genetic markers—microsatellite markers and chromosomal inversions—to unravel different evolutionary forces—which may shape the observed genetic variation—using estimates such as number of migrants per generation (*N_m_*), *F*‐statistics (*F_ST_*,* F_IS_*), and allele frequencies shifts.

## 2 MATERIALS AND METHODS

### 2.1 Sampling methods, geographic and climatic variables

We carried out 23 field trips to collect drosophilids from February 2005 to March 2011 in eight localities (Supporting Information Table S1). Five of them were near the boundary of the two GMRs (Figure 2). In the Peripheral Depression, we sampled in: Mata da Fazenda Santo Antonio, Capivari (CV); Mata Santa Genebra (SG); and Mata da Fazenda Santa Eliza, IAC, Costa e Silva, (CS), the first in the city of Capivari and the last two in the city of Campinas, all in São Paulo State. In the Atlantic Plateau, we sampled two forest remnants in the city of Campinas: Parque Ecológico Ms. Emílio José Salim (PE); and Mata do Ribeirão Cachoeira, Colinas do Atibaia (CA). We also sampled in eastern locations in the Atlantic Plateau: the Parque Nacional do Itatiaia (IT); and in Parque Nacional das Serra dos Órgãos, Teresópolis (TE), both in Rio de Janeiro State; and in the Reserva Municipal do Poço D'Anta, Juiz de Fora (JF) in Minas Gerais State. Populations from the boundary area (CV, SG, CS, PE, and CA) are in forest remnants with areas smaller than 255 ha (Table 1), we sampled them to assess differences linked to GMRs characteristics. Eastern Area samples were used since they live in bigger areas of continuous and well‐preserved forest, they probably suffer comparatively less effect of genetic drift than of natural selection. Populations were sampled, when possible, in different seasons trying to avoid biases due to seasonal differences in gene frequencies.

Actually, populations from CS, PE, CA, IT, and TE were sampled at least twice and in different seasons. PE and TE did not show a significant difference in inversion frequencies, after Bonferroni correction (*critical p* = 0.01), chi‐square values, respectively were as follows: PE: *χ* ^2^ = 5.06, *df*: 4; *p* = 0.281; and TE: *χ* ^2^ = 6.16, *df*: 2; *p* = 0.046. The other populations showed significant differences in their inversion frequencies (with Bonferroni correction): CS (*χ* ^2^ = 12.09, *df*: 3; *p* = 0.007), CA (*χ* ^2^ = 44.21, *df*: 20; *p* = 0.0014), and IT (*χ* ^2^ = 58.58; *df*: 14; *p* < 0.0001).

Costa e Silva population was sampled only twice and showed a clear difference in chromosome inversion frequencies between two consecutive years. So, it is highly desirable to have other samples from this fragment. However, despite the Administration efforts, the place has not been suitable for collections since 2008 due to security reasons beyond our possibilities. Thus, each sample was treated as a different population: CS07 and CS08.

In spite of the variations in chromosome inversion frequencies found in the other two populations (CA and IT), we decided to pool all the samples obtained for each population to carry out the statistical analyses since this seems to introduce a smaller a priori bias. Moreover, and most importantly, all the tests we used in the paper (see below) were robust. We repeated the tests using only cold‐dry season (Fall‐Winter) data, removing hot‐rainy season (Spring–Summer) data; furthermore, we also removed the data from 2010 and 2011, that were atypical years (due to the *La Niña* phenomenon). In all cases, for the statistical tests, we did for chromosome inversion frequencies the results remained qualitatively the same.

We collected drosophilids according to the proceedings described by Batista et al. (2018). Then, we brought the flies to laboratory and sorted them according to external morphology (Freire‐Maia & Pavan, 1949; Frota‐Pessoa, 1954). We crossed wild‐caught males individually with two or three virgin females from the homokaryotypic strain *ITC‐229‐ET*, routinely maintained in laboratory conditions (Carvalho, Peixoto, & Klaczko, 1989). We set up isofemale lines from wild females and used F_1_ male genitalia for species identification (we compared male F_1_ genitalia to drawings described by Frota‐Pessoa, 1954).

We obtained the geographical variables (latitude, longitude, altitude—Table 1) using a GPS device. We used data summaries of the nearest meteorological station (average monthly mean temperature and annual sum of monthly precipitation) for each site available at www.agritempo.gov.br (date of access: January 10, 2018) and www.ciiagro.sp.gov.br (date of access: January 10, 2018).

### 2.2 Cytological methods and chromosomal inversion frequencies

We prepared slides of polytene chromosomes following a protocol adapted from Ashburner (1989). First, we dissected 3rd instar larvae immersed in *Drosophila* Ringer solution. Then, salivary gland cells were fixed in a 1 N HCl solution with subsequent lacto‐acetic orcein staining for about 20 min. After that, we gently tapped the coverslip and squashed the slides. Finally, we observed chromosomes (inversion loops) under a microscope, identified landmarks such as polytene chromosome bands or puffs and compared them to chromosomal inversion breakpoints mapped in chromosome II (Ananina et al., 2002; Brianti et al., 2013).

We estimated chromosomal inversion frequencies using the “male” and “egg sample” methods (Ananina et al., 2004; Arnold, 1981). We inferred the wild male karyotype using up to eight F_1_ karyotyped larvae of the cross between the male and *ITC‐229‐ET* females. For the egg sample method, we karyotyped one isofemale F_1_ larva that we used for inferring the chromosomal inversion frequencies. We compared both estimated frequencies using a chi‐squared test (Zar, 2013), with not a single significant test. So, we pooled them and used the pooled frequencies for statistical analyses.

Ananina et al. (2004) pooled inversions *DS* and* DP*, because they have similar properties related to seasonality, temperature, precipitation, and altitude. Therefore, as they did, we grouped these inversions in our analysis. All other inversions (*DV*,* DJ*,* DT*,* DR*) were pooled as “Other” (*OT—*see in Bitner‐Mathé et al., 1995; Ananina et al., 2004).

### 2.3 Genetic and statistical analyses carried out using chromosomal inversion frequencies

We performed several analyses to characterize population divergence and their genetic structure revealed by inversion frequencies. First, we carried out an exploratory cluster analysis of the populations using Ward's algorithm method based on the minimum variance of Single Euclidian Distance of observed chromosomal inversion frequencies (Paradis, Claude, & Strimmer, 2004). This is not a test nor a phylogeny, its goal was to obtain a visual representation of the data.

To determine geographical and geomorphological influence on populations, we performed an independent test using the multiple matrix regression with randomization (MMRR). MMRR is essentially a multiple linear regression method applied to matrices (Wang, 2013). It may be used to quantify the association between distance matrices (such as geographic and environmental distances) and a dependent variable, such as genetic distances to make sure the results are not due to a statistical artifact. It is a method to disentangle the relative effects of isolation by distance (IBD) and isolation by environment (IBE), which does not suffer the limitations of the Partial Mantel test (Guillot & Rousset, 2013).

Then, to evaluate cluster heterogeneity, we used a chi‐squared test. We estimated hierarchical *F_ST_* of the inversion frequencies using Arlequin 3.5 (Excoffier & Lischer, 2010), this is similar to the analysis proposed by Ferrari and Taylor (1981). Populations were considered with low levels of population genetic structure when *F_ST_* < 0.05; moderate with 0.05 < *F_ST_* < 0.15; and *great* if *F_ST_* > 0.15 (Wright, 1951).

Finally, we tested the correlations between meteorological variables (average monthly mean temperature; annual sum of precipitation) versus genetic data (inversion frequencies after angular transformations Zar, 2013). We used the same procedure for geographical variables (latitude, longitude, altitude, logarithmic transformation of the square root of the area) versus genetic data.

### 2.4 Microsatellite genotyping

We also used the samples of six populations that were still available for microsatellite genotyping in the two distinct geomorphological regions (GMRs): three on the Atlantic Plateau—Teresópolis (TE *n* = 32 F_1_ females); Itatiaia (IT, *n* = 32 F_1_ females); Colinas do Atibaia (CA, *n* = 30 F_1_ females); and three on the Peripheral Depression—Capivari (CV, *n* = 32 F_1_ females); Santa Genebra (SG, *n* = 32 females from the field); and Costa e Silva (CS07, *n* = 34 wild males).

We extracted genomic DNA according to procedures described by Aljanabi and Martinez (1997). We used twelve microsatellite loci, which provided reliable genotyping, located in two inversion‐free chromosomes (five mapped on chromosome III—*Dmed*067 [locus name abbreviated from Dmed^UNICAMP^_ssr067; the other loci are similarly abbreviated henceforth]; *Dmed*085; *Dmed*087; *Dmed*096; *Dmed*106; and seven loci on chromosome V—*Dmed*011; *Dmed*025; *Dmed*028; *Dmed*053; *Dmed*072; *Dmed*098; *Dmed*119).

We used the forward primer for each locus labeled with a M13 fluorescent‐sequence (5′‐TGTAAAACGACGGCCAGT‐3′) at the 5′ end for genotyping (Schuelke, 2000). Polymerase chain reactions (PCRs) were set up in 5‐μl reaction volumes comprising 2.5 μl GoTaq Master Mix 2× (Promega), 0.5 μM of tag‐F primer +5 μM R‐primer mixture, 5 μM M13 primer, 50% glycerol (0.1 μl), 5 ng template DNA and 1.15 μl nuclease‐free water (Promega).

For sample amplification, we used a thermocycler (Veriti Thermal Cycler, Applied Biosystems): initial denaturation at 94°C for 4 min; followed by 10 cycles of 94°C/30 s, annealing temperature/1 min and 72°C/1 min; followed by 25 cycles of 89°C/30 s, annealing temperature/1 min, 72°C/1 min; and a final extension at 72°C/30 min. We resolved PCR products in an ABI PRISM 3,500‐XL automated sequencer (Applied Biosystems) using GeneScan 600 Liz (Applied Biosystems) as a molecular weight marker. We read genotypes using GeneMarker v.2.2 (SoftGenetics) with manual checking.

### 2.5 Genetic and statistical analyses carried out using microsatelites

We used GenAlEx 6.5 (Peakall & Smouse, 2012) to infer the estimates of genetic diversity, mean number of alleles per locus (*N_A_*), mean effective number of alleles (*N_E_*) and allele frequencies. Also, we estimated expected heterozygosity of microsatellite loci with Arlequin 3.5. (Excoffier & Lischer, 2010). We inferred null alleles influence in our samples, linkage disequilibrium and tested the alleles from all populations for deviations from Hardy–Weinberg equilibrium using software *Genepop on web* (Rousset, 2008). We obtained expected heterozygosity parameter from equation *H_e _=1−∑p_i_* ^2^, where *p_i_* ^2^ is the total expected frequency of the homokaryotypes for a chromosomal inversion.

We examined genetic structure population revealed by microsatellites using Wright's *F*‐statistics (Wright, 1951) estimated with *FSTAT* (Goudet, 1995) and through an analysis of molecular variance (AMOVA*‐*based on *F*‐statistics) with Arlequin 3.5 (Excoffier & Lischer, 2010), with 1,000 randomization and significant estimates considering *p* < 0.05. Similar to above, with chromosome inversions, we used the same criteria for determining the levels of population genetic structure. Then, we estimated the effective number of migrants per generation (*Nm*) by private allele method, implemented on *Genepop on web* (Rousset, 2008).

We also carried out an MMRR test, as we did with chromosome inversions, using Nei's Genetic Distance for the microsatellite data.

Finally, we tested the correlations between meteorological variables (average monthly mean temperature; annual sum of precipitation) versus genetic data (frequencies of three most common alleles and expected heterozygosity [*H_e_*] after angular transformations Zar, 2013). We used the same procedure for geographical variables (latitude, longitude, altitude, logarithmic transformation of the square root of the area) versus genetic data.

## 3 RESULTS

### 3.1 Population genetic structure revealed by chromosomal inversions

**Figure 3:**
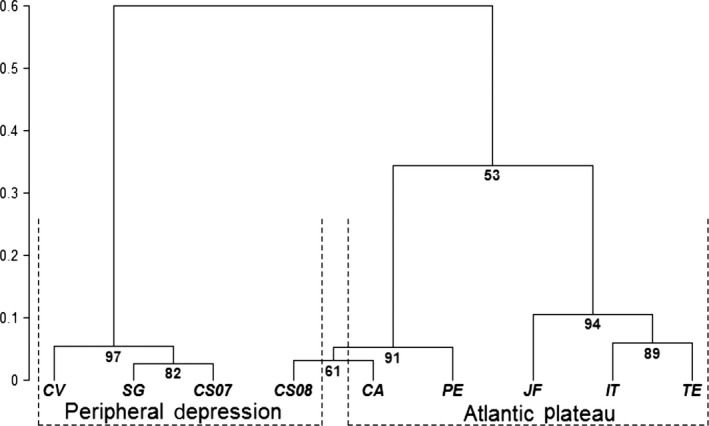
Dendrogram of the Clustering Analysis using Ward´s algorithm method based on Single Euclidian Distance from the observed frequencies of chromosome II inversions (CV: Capivari; SG: Mata Santa Genebra; CS07 and CS08: Costa e Silva, 2007 and 2008 samples; PE: Parque Ecológico; CA: Colinas do Atibaia; IT: Itatiaia; JF: Juiz de Fora; TE: Teresópolis). Please note, this is not a phylogeny nor a test, it is just a similarity dendrogram

We observed three distinct groups in the cluster analysis (Figure 3): cluster 1*—*Capivari (CV), Santa Genebra (SG), and Costa e Silva 2007 (CS07); cluster 2—Parque Ecológico (PE), Colinas do Atibaia (CA), Costa e Silva 2008 (CS08); cluster 3—Juiz de Fora (JF), Itatiaia (IT), and Teresópolis (TE). The last two groups are separated from the first, but this division has no support. Nevertheless, they reflect quite well their location in the two geomorphological regions of the area: Peripheral Depression and Atlantic Plateau; with the exception of the Costa e Silva population (CS07 and CS08). In 2007 (CS07), this population grouped with cluster 1; and in 2008 (CS08), it grouped with cluster 2 (Figure 3).

Multiple matrix regression with randomization analysis showed a clear result when examining the spatial variation of chromosome inversion frequencies. We observed that geographical (geodesic) and environmental (GMRs) distances were significantly associated with chromosomal variation (*R* ^2^ = 0.679; *F* = 34.97; *df:* 1, 9; *p* = 0.001; Intercept: −0.008, *p* = 0.989; βGEOG = 0.596, *p* = 0.001; βENV = 0.454, *p* = 0.003). These estimates showed that even controlling the geographical distance, the environmental factor (geomorphological region—GMR) was still significant.

Pairwise genetic distances indicated that populations grouped in cluster 1 were less different than others populations within cluster 2 (Supporting Information Table S3). Their genetic distances varied between 0.004 and 0.013, while in the clade with clusters 2 and 3 the distances varied between 0.006 and 0.793. CV and JF were the most distant populations (their distance was 0.793); while SG and CS07 showed the lowest genetic distance (0.004).

Inversion frequency estimates for all populations showed consistent patterns with cluster analysis (Table 2). Populations of Parque Ecológico (PE) and Colinas do Atibaia (CA), which lie in the Atlantic Plateau showed DS+DP in frequencies under 30%. Populations from Capivari (CV) and Santa Genebra (SG), both in the Peripheral Depression, with similar frequencies showed DS+DP frequencies above 50%. When we tested all populations (ignoring the clusters), we found they were highly significantly heterogeneous (*χ* ^2^ = 370.3; *df *= 32; *p* < 0.0001). Cluster 1 grouped only populations from Campinas Area which lied in Peripheral Depression (CV, SG, CS07) and they were not heterogeneous (*χ* ^2^ = 4.5; *df* *=* 8; *p* > 0.8). Similarly, cluster 2 is not heterogeneous (*χ* ^2^ = 13.4; *df* *=* 8; *p* > 0.1). However, populations only in forest fragments near Campinas area (clusters 1 and 2) were highly significantly heterogeneous (*χ* ^2^ = 98.6; *df* *= *20; *p* < 0.0001). The clade with cluster 2 and cluster 3 which grouped some populations from Campinas Area (CS08, PE, and CA) and from Eastern Samples (IT, JF, and TE) was highly heterogeneous (*χ* ^2^ = 180.04; *df* = 10; *p* < 0.001); although cluster 3 is also heterogeneous (*χ* ^2^ = 16.9; *df* = 4; *p* < 0.01).

**Table 2:** Inversion frequency distribution (values expressed as percentage; OT: other rare inversions; 2N: number of chromosomes analyzed)

Population	DA	DI	DS +DP	OT	2N
CV	25.00	6.48	56.02	12.50	216
SG	25.68	10.81	50.00	13.51	74
CS07	26.83	9.76	46.34	17.07	82
CS08	43.69	14.41	33.33	8.57	222
PE	41.18	17.65	24.79	16.38	238
CA	43.57	14.56	28.88	12.99	824
IT	50.00	29.52	14.84	5.64	620
JF	41.38	43.10	5.17	10.35	58
TE	55.85	35.11	6.91	2.13	188
Total					2,522

Note

CV: Capivari; SG: Santa Genebra; CS: Costa e Silva; PE: Parque Ecológico; CA: Colinas do Atibaia; IT: Itatiaia; JF: Juiz de Fora; TE: Teresópolis.

Hierarchical *F_ST_* analysis of chromosomal inversion genetic distance revealed that more than 91% of the total variation was among individuals within populations with moderate levels of genetic structure among all populations (*F_ST_* = 0.088; *p* = 1 × 10^−5^). AMOVA also showed that 5% of the total variation can be explained by the structuration between the GMRs (*F_RT_* = 0.049), while 4% of total variation explained by the structuration among populations within GMRs (*F_PR_* = 0.041—Table 3).

**Table 3:** Hierarchical analyses of molecular variance (AMOVA) with fixation indexes due to differences: between geomorphological regions (GMRs; *F_RT_*); among populations within GMRs (*F_PR_*); and among populations in the total (*F_ST_*); and components of the total genetic variance (in percentage) for: between GMRs; within GMRs; and within all populations. AMOVAs estimated using: *Inv*—chromosomal inversion frequencies; and *ssr*—microsatellite loci

Marker	Fixation index		
	*F_RT_*	*F_PR_*	*F_S__T_*
*Inv*	0.049 (*p = *0.012 ± 0.003)	0.041 (*p = *0.0000 ± 0.000)	0.088 (*p = *0.0000 ± 0.000)
*ssr*	0.006 (*p = *0.395 ± 0.0046)	0.016 (*p = *0.0000 ± 0.000)	0.022 (*p = *0.0000 ± 0.000)

Marker	Variance		
	Between GMRs (%)	Among populations within GMRs (%)	Within populations (%)
*Inv*	4.97	3.86	91.17
*ssr*	0.60	1.58	97.82

### 3.2 Clines and correlations of chromosomal inversions polymorphism

We examined the correlations between inversion frequencies and latitude, longitude, altitude, area, average monthly mean temperature, and annual sum of monthly precipitation of the collecting sites (Table 4). We observed significant correlations between three meteorological and geographical variables (temperature, longitude, and altitude) versus the frequencies of all inversions. Inversions DI, DS+DP still showed significant correlation with latitude (*r* *=* −0.91, *p* = 0.001; *r = *0.82, *p* = 0.006; respectively), while OT was correlated with area (*r* *= *−0.82, *p* = 0.007). However, after applying Bonferroni procedure, only correlations between latitude, longitude, and altitude with inversions DI and DS+DP remained significant. Finally, inversions DA and DI showed similar patterns in their correlations with meteorological and geographical variables, while DS+DP showed the opposite pattern. For example, the correlations of DA and DI with temperature were, respectively, *r* *= *−0.81, *p* = 0.009; and *r* *= *−0.86, *p* = 0.003; while, DS+DP was *r = *0.86, *p = *0.003. However, when Bonferroni procedures are applied, no correlation with temperature remained significant.

**Table 4:** Pearson's Correlations (*r*) between inversion on distal region of chromosome II frequencies (after angular transformation) versus latitude, longitude, altitude, area, average monthly mean temperature, and annual sum of monthly precipitation

Variables	DA	DI	DS + DP	OT
Latitude	−0.44*^ns^*	−0.91^***^	0.82^**^	0.42*^ns^*
Longitude	−0.69^*^	−0.95^***^	0.91^***^	0.76^*^
Altitude	0.68^*^	0.98^***^	−0.96^***^	−0.51*^ns^*
Area	0.62*^ns^*	0.65*^ns^*	−0.61*^ns^*	−0.82^**^
Temperature	−0.81^**^	−0.86^**^	0.86^**^	0.70^*^
Precipitation	0.46*^ns^*	0.39*^ns^*	−0.39*^ns^*	−0.19*^ns^*

Note

ns: non‐significant; *p < 0.05; **p < 0.01; ***p < 0.001.

We also tested independently the correlations between frequencies of inversions in the second chromosome proximal region, using only wild male karyotypes, with the same meteorological and geographical variables mentioned above (data not shown). The general pattern was consistent with the distal inversions mentioned above.

### 3.3 Effects of forest fragmentation on chromosomal inversion polymorphism

Populations from *Costa e Silva* (CS07 and CS08), Parque Ecológico (PE) and Colinas do Atibaia (CA) are distant less than 20 km from each other. We sampled these populations in different occasions (twice for CS; twice for PE; and six times CA) to test their genetic resilience (chromosomal inversion frequency resilience). For each one, we performed a contingency chi‐squared test to detect variation among collections and no significant differences were observed for PE and CA populations (respectively,* χ* ^2^ = 3.97; *df* = 4; *p* > 0.5; *χ* ^2^ = 24.97; *df* = 20; *p* > 0.25). However, CS07 and CS08 were statistically different (*χ* ^2^ = 9.39, *df *= 4; *p* < 0.05).

### 3.4 Population genetic structure revealed by microsatellites

We observed great variability of microsatellite markers (Table 5), with 312 distinct alleles considering all 12 analyzed loci. Among them, 85 were private alleles and 227 shared between two or more populations. Population from SG showed the highest mean number of alleles (*N_A_* = 15.2) and private alleles (*P_A_* = 2.2), while TE population showed the smallest mean number of alleles (*N_A_* = 13.3) and of private alleles (*P_A_* = 0.6). Mean expected heterozygosity (*H_e_*) ranged from 0.82 in IT to 0.86 in SG.

**Table 5:** Estimates of microsatellite‐based genetic diversity of six *Drosophila mediopunctata* population. *N*: sample size per population; *N*_A_: mean number of alleles; *P*_A_: mean number of private alleles; *N*_E_: mean effective number of alleles; *H_e_*: expected heterozygosity

Populations	*N*	*N*_A_	*P*_A_	*N*_E_	*H_e_*
CV	32	14.75	1.33	7.35	0.83
SG	32	15.17	2.17	7.81	0.86
CS07	34	13.42	1.08	6.93	0.84
CA	30	13.75	0.92	7.13	0.83
IT	32	14.50	1.00	7.39	0.82
TE	32	13.25	0.58	6.65	0.83

Note

CV: Capivari; SG: Santa Genebra; CS07: Costa e Silva; CA: Colinas do Atibaia; IT: Itatiaia; TE: Teresópolis.

We analyzed linkage disequilibrium (*LD*) between pairs of loci for every population. Among 31 associations, only four remained significant after applying Bonferroni procedures: three in TE population (between loci *Dmed*096‐*Dmed*106 on chromosome III; between loci *Dmed*011‐*Dmed*072 and *Dmed*028‐*Dmed*072 on chromosome V) and one association in SG population (on chromosome V, between loci *Dmed*098*‐Dmed*119).

We estimated inbreeding coefficient (*F_IS_*) values for every locus and every population (Table 6). We observed that SG population showed ten loci with significant deviations (*Dmed*028, *Dmed*053, *Dmed*067, *Dmed*072, *Dmed*085, *Dmed*087, *Dmed*098, *Dmed*106, and *Dmed*119). Population CS07 showed eight loci (*Dmed*053, *Dmed*067, *Dmed*072, *Dmed*085, *Dmed*098, *Dmed*106, and *Dmed*119). TE showed six loci (*Dmed*011, *Dmed*025, *Dmed*028, *Dmed*067, *Dmed*096, and *Dmed*119). Populations CA (*Dmed*028, *Dmed*067, *Dmed*098, and *Dmed*119) and CV (*Dmed*085, *Dmed*098, *Dmed*106, and *Dmed*119) showed four loci. Finally, Itatiaia showed only one locus with deviation (*Dmed*119). Out of 72 tests, only one locus (*Dmed*119) showed deviation of Hardy–Weinberg equilibrium in all six populations, after applying sequential Bonferroni procedures.

**Table 6:** Loci inbreeding index (*F_IS_*) inferred for six *Drosophila mediopunctata* population

Loci	CV	SG	CS07	CA	IT	TE	Mean
*Dmed*011	0.03	0.16	−0.01	0.01	−0.04	0.14	0.05
*Dmed*025	0.04	0.07	0.10	0.14	0.19	0.28	0.14
*Dmed*028	0.14	0.21	0.23	0.13	0.10	0.19	0.17
*Dmed*053	0.16	0.27	0.42	0.09	0.06	0.27	0.22
*Dmed*067	0.12	0.20	0.51	0.20	−0.05	0.38	0.24
*Dmed*072	0.12	0.27	0.28	0.06	0.03	0.03	0.13
*Dmed*085	0.21	0.30	0.21	0.17	0.21	0.31	0.24
*Dmed*087	−0.17	0.02	0.13	0.31	−0.04	0.10	0.06
*Dmed*096	0.12	0.44	0.46	0.10	0.08	0.08	0.22
*Dmed*098	0.05	0.26	0.11	0.07	0.09	0.00	0.09
*Dmed*106	0.20	0.08	0.26	0.27	0.12	0.15	0.18
*Dmed*119	0.39	0.19	0.11	0.28	0.45	0.23	0.28
Average	0.12	0.21	0.23	0.15	0.10	0.18	0.17

Note

CV: Capivari; SG: Santa Genebra; CS07: Costa e Silva; CA: Colinas do Atibaia; IT: Itatiaia; TE: Teresópolis.

We found relatively low levels of population genetic structure (overall *F_ST_* estimate of microsatellite markers was *F_ST_* = 0.022; *p* < 0.0001) among these six populations. We estimated genetic differentiation between pairs of populations (pairwise *F_ST_* analysis of microsatellites genetic distance—Table 7). Only four out of 15 comparisons were not significant. The smallest difference was between CS07 and SG as well as between IT and CV (*F_ST_* = 0.003*^ns^*; ns: non‐significant), while populations most genetically distant were CS07 and CV (*F_ST_* = 0.036; *p < *0.00001). We observed no significant differences among hierarchical levels microsatellite‐based AMOVA (Table 3). Variation within populations explained about 98% of total variation. On the other hand, only 0.6% of the total variation is due to the variation between the different GMRs and is non‐significant (*F_RT_* = 0.006*^ns^*).

**Table 7:** Population pairwise *F*_ST_ and number of migrants (*Nm* using the method of microsatellite private alleles)

Between populations	*F_ST_*	*Nm*
SG × CV	0.024***	2.6
CS07 × CV	0.036***	2.9
CS07 × SG	0.003*^ns^*	5.8
CA × CV	0.004*^ns^*	6.2
CA × SG	0.024***	3.0
CA × CS07	0.030***	2.4
IT × CV	0.003*^ns^*	5.3
IT × SG	0.024***	2.8
IT × CS07	0.034***	3.0
IT × CA	0.004*^ns^*	6.7
TE × CV	0.015***	3.5
TE × SG	0.025***	3.4
TE × CS07	0.031***	3.1
TE × CA	0.011*	3.3
TE × IT	0.017***	4.1

Note

ns, non‐significant; *p < 0.05; **p < 0.01; ***p < 0.001

CV: Capivari; SG: Santa Genebra; CS07: Costa e Silva; CA: Colinas do Atibaia; IT: Itatiaia; TE: Teresópolis.

Multiple matrix regression with randomization analysis for microsatellite data detected no effect of spatial variation (*r* ^2^ = 0.0017; *p = *0.993). We observed that geographical (geodesic) and environmental (geomorphological region) distances were not associated with microsatellite both for geodesic distance (βGEOG) and ecological distance (βENV; Intercept: 0.3492; *p* = 0.362; βGEOG = −0.0301; *p* = 0.963; βENV = −0.0476; *p* = 0.901). These estimates show a clear contrast with the chromosome inversion data.

In addition, we tested the correlation between meteorological and geographical variables of the collecting sites with the three most frequent alleles for each locus. Among the twelve loci studied, only two showed significant correlations with any geographical variation. The third most common allele of locus *Dmed*028 showed a significant positive correlation with latitude and temperature (*r* = 0.83; *p* = 0.04 and *r = *0.88; *p* = 0.02, respectively) along a significant negative correlation with altitude, area, and precipitation (*r* = −0.95; *p* = 0.003; *r* = −0.90; *p* = 0.014; and *r* = −0.86; *p* = 0.030; respectively). The second most common allele of locus *Dmed*087 showed the opposite pattern, a significant positive correlation with altitude, area, and precipitation (*r* = 0.84; *p* = 0.038; *r* = 0.95; *p* = 0.004; and *r* = 0.91; *p* = 0.013; respectively) along a significant negative correlation with latitude and temperature (*r* = −0.84; *p* = 0.036 and *r* = −0.93; *p* = 0.008; respectively). However, after the application of Bonferroni procedure, no correlation remained significant.

The estimated number of migrants (using private alleles method) among all populations was *Nm* = 7.9; while using the *F_ST_* method was *Nm = *11.7. The lowest *Nm* value found was between CA and SG (*Nm* = 2.4)—populations distant 15 km from each other—while the highest was between IT and CA (*Nm *= 6.7)—distant 267 km from each other. Geographically the closest populations are CS and SG with *Nm = *5.8, while the most distant are TE and CV with *Nm* = 3.5. In any case, the number of migrants per generation is high.

## 4 DISCUSSION

Apart from some beneficial effects over bird guilds (Terraube et al., 2016), habitat loss and forest fragmentation are expected to generate small‐scale environmental heterogeneity as well as to affect natural populations by drastic reduction of their population size and gene flow (Haddad et al., 2015). These demographic effects can be characterized using genetic markers to assess the genetic structure, effective population size, linkage disequilibrium, dispersion pattern, and changes in allele frequencies (Keyghobadi, 2007).

*F*‐statistics analyses revealed excesses of homozygotes for microsatellites in most samples, with significant *F_IS_.* This, probably, is due to an artifact caused by the possible presence of null alleles (Supporting Information Table S2 shows null alleles frequencies per locus in all populations). Some population parameters such as *F_IS_* and *expected homozygosity* may be biased by the presence of null alleles (Chapuis & Estoup, 2007; Waples, 2018). However, loci with null alleles did not show a marked loss in genetic diversity; besides, out of 31 possible linkage disequilibria between loci, only four remained significant after Bonferroni procedures. Our results do not show any indication of drastic genetic losses associated with bottleneck events. Moreover, despite the presence of some null alleles, there is clear evidence for important genetic variation. This suggests that fragmentation effects are not particularly impacting in this species (at least presently)—this is also reinforced by the low population structure observed.

Our results indicate an overall moderate genetic structure for chromosomal inversions and low genetic structure for microsatellite loci. It is noteworthy that overall *F_ST_* revealed by chromosomal inversions (0.088) is four times higher than *F_ST_* revealed by microsatellites (0.022). Dissecting this genetic structure, AMOVA (Table 3) shows that differentiation between the two GMRs for chromosomal inversions (*F_RT_* = 0.049) is eight times the non‐significant differentiation found with microsatellites (*F_RT_* = 0.006).

The patterns for population structure indicate that the chromosomes may be subject to evolutionary forces of different magnitude. Furthermore, the observed differences in chromosomal inversion polymorphism may be liable to local differentiation, reflecting the action of natural selection.

Schiffer, Kennington, Hoffmann, and Blacket (2007) observed low levels of genetic structuring caused by the fragmentation process, indicating strong influence of migration among Australian populations of *Drosophila birchii*. Urban and non‐urban populations of *Drosophila subobscura* from Serbia had their genetic structure examined, using chromosome inversions as markers. Despite a strong anthropogenic influence on the population from Belgrade, it did not show any loss in its inversion polymorphisms, as well as in its population size; therefore, this population does not seem to suffer the negative effects caused by human activity and urbanization (Kenig, Jelic, Kurbalija, Stamenkovic‐Radak, & Andjelkovic, 2010). Their results suggest that divergence in chromosome inversion polymorphisms among Serbian populations of *D. subobscura* may be an adaptive response to differences among environments they live in (Stamenković‐Radak et al., 2012).

Divergence among *D. mediopunctata* populations may be more associated with climatic and geomorphological properties of both regions than to harmful effects of forest fragmentation. Similarly, divergences in species diversity for Ithomiinae butterflies sampled in the same GMRs may be correlated to geomorphological differences (Brown & Freitas, 2000; 2002).

Overall, populations can be grouped in three different clusters according to their genetic distance and their geomorphological location (Figure 3). The only exception is the population from Costa e Silva, which grouped within clusters 1 and 2 in successive collecting years. The variation in frequencies observed between two consecutive years may be interpreted as result of migration from adjacent areas, since it is located near the border of the two GMR. However, it also could be caused by drastic changes in thermal regime associated with forest fragmentation process—the monthly average temperature for April 2007 was 24.1°C; while for June 2008 was 18.8°C. This difference in thermal regime may induce a seasonal cycle of inversion DA, since this inversion showed a seasonal cycle in IT population since the 1980s (Ananina et al., 2004; Batista et al., 2012).

Ananina et al. (2004) previously showed, in samples from the 80 s, significant correlations with the average temperature of the collecting month for the frequencies of inversions DA (negative; *r = *−0.91) and DS+DP (positive; *r = *0.67). Now, we observed similar correlation values for the same arrangements and average annual temperature of each location—negative for DA (*r = *−0.81) and positive for DS+DP (*r = *0.86). Surprisingly, we found a strong and significant correlation between *DI* and longitude (*r = *− 0.95) as shown in Table 4. This gene arrangement also showed significant correlation with altitude in samples of 2007–2010 from the Itatiaia population (Batista et al., 2012). The biological meaning of this correlation is an open question that should be further analyzed, perhaps involving biotic factors.

Variations in genetic polymorphisms concomitant with environmental gradients can be considered signs of local adaptation which may lead to population divergence (Wellenreuther et al., 2017). We could not find a consistent pattern of geographical variation for any microsatellite locus. In contrast, inversion polymorphism showed clinal variations congruent with previous findings (Ananina et al., 2004; Batista et al., 2012). This highlights that for a complete understanding of how the fragmentation process is affecting a species, studies should be carried out using different genetic markers to evaluate the joint effects of natural selection, migration and genetic drift.

In summary, our results suggest that, with few exceptions, differences in inversion frequencies of fragmented populations can be maintained according to their geomorphological origin in spite of the effects of gene flow and genetic drift.

## CONFLICT OF INTEREST

None.

## AUTHOR CONTRIBUTIONS

Marcos R.D. Batista conceived general project, participated of all collecting occasions, sorted and karyotyped *Drosophila mediopunctata* natural populations, analyzed the data, wrote the paper, prepared figures and/or tables, reviewed drafts of the paper. Rafael E. S. Penha carried out microsatellite genotyping and genetic structure analysis, wrote the paper, prepared figures and/or tables, reviewed drafts of the paper. Silvia H. Sofia contributed with reagents/materials/analysis tools, wrote the paper, and reviewed drafts of the paper. Louis B. Klaczko participated of all collecting occasions, analyzed the data, contributed reagents/materials/analysis tools, wrote the paper, prepared figures and/or tables, reviewed drafts of the paper, conceived general project.

## DATA ACCESSIBILITY

Data available at Dryad: https://doi.org/10.5061/dryad.9r4r7p1.
