# Supplementary material for: Comparative analysis of adaptive and neutral markers of Drosophila mediopunctata populations dispersed among forest fragments
Source: Ecol Evol. 2018 Nov 22;8(24):12681–93. doi: 10.1002/ece3.4696 (PMC6308856; doi:10.1002/ece3.4696)
Supplement: Supplementary file 1 [file ECE3-8-12681-s001.docx]

**Table S1**. Field work dates and localities (**GMR**: geomorphological region; **N**: number of individuals analyzed).

| **GMR** | **Local** | **Collection Date** | **N** |
| --- | --- | --- | --- |
| **Peripheral Depression** | Capivari (*CV*) | October 14-16, 2008. | 108 |
|  | Santa Genebra (*SG*) | July 22-24, 2008. | 39 |
|  | Costa e Silva (*CS*) | April, 23-27, 2007. | 41 |
|  |  | June 3-6, 2008. | 111 |
| **Atlantic Plateau** | P. Ecológico (*PE*) | October 4-11, 2006. | 19 |
|  |  | May 26-28, 2008. | 100 |
|  | Colinas do Atibaia (*CA*) | February 23-25, 2005. | 69 |
|  |  | September 28 – October 2, 2005. | 59 |
|  |  | August 21-23, 2008. | 84 |
|  |  | March 16-20, 2010. | 64 |
|  |  | August 31 – September 4, 2010. | 43 |
|  |  | March 21-24, 2011. | 81 |
|  | P. N. Itatiaia (*IT*) | June 1-4, 2007. | 48 |
|  |  | February 22-26, 2008. | 30 |
|  |  | November 19-24, 2008. | 74 |
|  |  | March 10-16, 2009. | 51 |
|  |  | May 30 – June 5, 2009. | 53 |
|  |  | March 3-10, 2010. | 26 |
|  |  | August 23-27, 2010. | 66 |
|  |  | March 21-24, 2011. | 19 |
|  | Juiz de Fora (*JF*) | July 4-5, 2009. | 29 |
|  | Teresópolis (*TE*) | August 11-14, 2009. | 64 |
|  |  | September 24-27, 2009. | 26 |

**Table S2**. Frequency of null alleles per loci and population.

| **Loci** | **TE** | **IT** | **CA** | **CS** | **SG** | **CV** | **Average** |
| --- | --- | --- | --- | --- | --- | --- | --- |
| ***Dmed*011** | 0.05 | 0.00 | 0.02 | 0.01 | 0.05 | 0.00 | 0.02 |
| ***Dmed*025** | 0.12 | 0.04 | 0.07 | 0.06 | 0.00 | 0.00 | 0.05 |
| ***Dmed*028** | 0.09 | 0.05 | 0.03 | 0.11 | 0.09 | 0.04 | 0.07 |
| ***Dmed*053** | 0.11 | 0.03 | 0.04 | 0.21 | 0.12 | 0.05 | 0.09 |
| ***Dmed*067** | 0.16 | 0.00 | 0.05 | 0.21 | 0.08 | 0.07 | 0.10 |
| ***Dmed*072** | 0.01 | 0.00 | 0.02 | 0.13 | 0.13 | 0.06 | 0.06 |
| ***Dmed*085** | 0.15 | 0.10 | 0.10 | 0.12 | 0.14 | 0.10 | 0.12 |
| ***Dmed*087** | 0.02 | 0.00 | 0.12 | 0.05 | 0.04 | 0.00 | 0.04 |
| ***Dmed*096** | 0.02 | 0.04 | 0.03 | 0.21 | 0.20 | 0.05 | 0.09 |
| ***Dmed*098** | 0.00 | 0.03 | 0.00 | 0.04 | 0.11 | 0.02 | 0.03 |
| ***Dmed*106** | 0.06 | 0.04 | 0.13 | 0.14 | 0.05 | 0.08 | 0.08 |
| ***Dmed*119** | 0.11 | 0.21 | 0.14 | 0.13 | 0.11 | 0.17 | 0.14 |
| **Average** | 0.07 | 0.04 | 0.06 | 0.12 | 0.09 | 0.05 | **0.07** |

(**CV**: Capivari; **SG**: Santa Genebra; **CS07**: Costa e Silva; **CA**: Colinas do Atibaia;
 **IT**: Itatiaia; **TE**: Teresópolis).

Table S3 – Genetic and Geographical distances between populations. Lower diagonal shows Nei’s genetic distances estimated with chromosomal inversions frequencies on *Gendist*, a program from the PHYLIP 3.7 package (Felsenstein, 1993). Upper diagonal shows geographical distances (expressed in km).

|  | **CV** | **SG** | **CS 07** | **CS 08** | **PE** | **CA** | **IT** | **JF** | **TE** |
| --- | --- | --- | --- | --- | --- | --- | --- | --- | --- |
| **CV** | 0 | 47 | 44 | 44 | 56 | 65 | 331 | 491 | 508 |
| **SG** | 0.005 | 0 | 7 | 7 | 15 | 21 | 288 | 446 | 467 |
| **CS 07** | 0.013 | 0.004 | 0 | 0 | 9 | 15 | 283 | 441 | 461 |
| **CS 08** | 0.135 | 0.099 | 0.087 | 0 | 9 | 48 | 283 | 441 | 461 |
| **PE** | 0.212 | 0.154 | 0.125 | 0.022 | 0 | 14 | 279 | 438 | 457 |
| **CA** | 0.170 | 0.125 | 0.103 | 0.006 | 0.007 | 0 | 267 | 426 | 446 |
| **IT** | 0.480 | 0.380 | 0.352 | 0.092 | 0.062 | 0.077 | 0 | 165 | 181 |
| **JF** | 0.793 | 0.619 | 0.578 | 0.262 | 0.169 | 0.224 | 0.052 | 0 | 94 |
| **TE** | 0.718 | 0.579 | 0.544 | 0.177 | 0.132 | 0.155 | 0.013 | 0.040 | 0 |
